# Degradation profile of nixtamalized maize pericarp by the action of the microbial consortium PM-06

**DOI:** 10.1186/s13568-019-0812-7

**Published:** 2019-06-13

**Authors:** José Germán Serrano-Gamboa, Rafael Antonio Rojas-Herrera, Araceli González-Burgos, Jorge Luis Folch-Mallol, Diego Javier Jiménez, Mónica Noel Sánchez-González

**Affiliations:** 1 Facultad de Ingeniería Química, Universidad Autónoma de Yucatán, Campus Ciencias Exactas e Ingenierías, Periférico Norte, Kilómetro 33.5, Tablaje Catastral 13615, Chuburná de Hidalgo Inn, Mérida, Yucatán Mexico; 2 Centro de Investigación en Biotecnología, Universidad Autónoma del Estado de Morelos, Cuernavaca, Mexico; 3 Microbiomes and Bioenergy Research Group, Department of Biological Sciences, Universidad de los Andes, Bogotá, Colombia

**Keywords:** Nixtamalized maize pericarp, Endogenous microbial consortium, Lignocellulose degradation mechanism, Xylanase and cellulase activities

## Abstract

The nixtamalized maize pericarp (NMP) is a plentiful by-product of the tortilla industry and an important source of fermentable sugars. The aim of this study was to describe the degradation profile of NMP by the action of a consortium (PM-06) obtained from the native microbial community of this residue. The degradation was analyzed in terms of the changes in the community dynamics, production of enzymes (endo-xylanase and endo-cellulase), physicochemical parameters, and substrate chemical and microstructural characteristics, to understand the mechanisms behind the process. The consortium PM-06 degraded 86.8 ± 3.3% of NMP after 192 h of growth. Scanning electron microscopy images, and the composition and weight of the residual solids, showed that degradation was sequential starting with the consumption of hemicellulose. Xylanase was the highest enzyme activity produced, with a maximum value of 12.45 ± 0.03 U mL^−1^. There were fluctuations in the pH during the NMP degradation, starting with the acidification of the culture media and finishing with a pH close to 8.5. The most abundant species in the consortium, at the moment of maximum degradation activity, were *Aneurinibacillus migulanus, Paenibacillus macerans, Bacillus coagulans, Microbacterium* sp*. LCT*-*H2*, and *Bacillus thuringiensis.* The diversity of PM-06 provided metabolic abilities that in combination helped to produce an efficient process. The consortium PM-06 generated a set of different tools that worked coordinated to increase the substrate availability through the solubilization of components and elimination of structural diffusion barriers. This is the first report about the degradation of NMP using a microbial consortium.

**Electronic supplementary material:**

The online version of this article (10.1186/s13568-019-0812-7) contains supplementary material, which is available to authorized users.

## Introduction

Agro-industrial residues are important sources of fermentable sugars useful for the production of biofuels, biomaterials and biocatalysts (Bhatia et al. 2012). However, the structural complexity makes the sustainable use of these residues a technological challenge. In Mexico and Central America, maize is nixtamalized before consumption (Serna-Saldivar 2016). This process, involves the alkaline cooking and steeping of maize grains in a lime solution. Nixtamalized maize is used for the production of different staples within the Mexican diet, such as “tortillas”. Millions of tons of maize are processed annually, generating significant amounts of by-products including nixtamalized maize pericarp (NMP) (Campechano-Carrera et al. 2012; García-Zamora et al. 2015). Nixtamalization modifies the external layers of pericarp and promotes the partial solubilization of hemicellulose, without affecting the structure of the internal tissue (Gutiérrez-Cortez et al. 2010). The integrity of pericarp after nixtamalization, depends on the processing conditions (time and temperature of cooking and alkali concentration) (Gutiérrez-Cortez et al. 2010). Industrial NMP is a residue rich in hemicellulose (~ 45%), followed by cellulose (~ 19%), starch (~ 13%) and lignin (~ 5%) (Rostro-Alanís et al. 2014). Like other recalcitrant lignocellulosic structures, NMP requires the use thermochemical (207 °C) or harsh alkaline treatments to recover high valuable components such as hemicellulose (López-Martínez et al. 2011; Rostro-Alanís et al. 2014).

In nature, microbial communities decompose plant polysaccharides by the synergistic action of thousands of species (Jose et al. 2017; Tláskal et al. 2017). The selection of lignocellulolytic microbial consortia has been considered a promising approach to improve the saccharification of agricultural residues (Carlos et al. 2018; Cortes-Tolalpa et al. 2016; Du et al. 2015). In consortia, microorganisms co-exist, secrete an array of enzymes, and act in synergic ways to efficiently release sugars from plant polysaccharides. Moreover, the culture conditions, microbial origin, type of substrate and the dynamic of conditions present in the system, are main drivers of the diversity and metabolic potential in a lignocellulose degrading consortium (Jiménez et al. 2017; Zhu et al. 2016). Therefore, lignocellulose degradation by consortia is multifactorial and must be analyzed in a holistic way to understand the mechanisms involved.

The high content of organic matter present in the nixtamalization residues, suggests the abundant presence of microorganisms (Sanchez-Gonzalez et al. 2011). However, the microbiota of these residues and their potential uses have not been fully explored. *Paenibacillus amyloliticus*, *Pseudomonas putida* and *Acinetobacter* were isolated from soil contaminated with nixtamalization liquors (nejayote) (Salmeron-Alcocer et al. 2003). These microorganisms are known as degraders of lignocellulose components or members of lignocellulose degrading consortia (Auer et al. 2017; Keggi and Doran-Peterson 2019; Ravi et al. 2017). Moreover, strains of *Bacillus flexus* isolated from nejayote were able to release phenolic acids from NMP (Sanchez-Gonzalez et al. 2011). These evidences suggest the potential ability of the microorganisms present in the nixtamalization residues to degrade lignocellulose. In this work is described the degradation profile of NMP by the action of the consortium PM-06, obtained from the native microbial community of this residue. The degradation was analyzed in terms of the changes in the community dynamics, production of enzymes (endo-xylanase and endo-cellulase), physicochemical parameters, and substrate chemical and microstructural characteristics, to understand the mechanisms behind the process.

## Materials and methods

### Origin and selection of the native microbial consortium PM-06

Consortium PM-06 was enriched from the endogenous microbiota in NMP by the dilution-to-stimulation approach (Lee et al. 2013). Cell suspensions of the NMP microbiota were obtained according to De Lima Brossi et al. (2016). Aliquots of 250 µL of this cell suspension were added to 25 mL of a medium containing 40 g NMP L^−1^ (Harinera de Yucatán, S.A. de C.V., Mérida, México) and 5 g yeast extract L^−1^, and incubated at 37 °C and 125 rpm for 7 days. After the incubation time, 2 mL of these cultures were used to inoculate fresh media. This procedure was repeated until the functional stability was maintained. The functional stability was determined measuring the amount of residual NMP present in the culture after 168 h. All experiments were conducted in triplicate. The composition of the NMP lot used was: 15.3% cellulose, 23.7% xylan, 12.6% arabinose substituents, 33% starch, 4.4% lignin, and 11% of others components (ashes, extractives, uronic acids and protein).

### Consortium PM-06 culture conditions for the degradation of NMP

The microbial consortium PM-06 was cultivated in 25 mL of media containing 40 g dried NMP L^−1^ and 5 g yeast extract L^−1^. These flasks were inoculated with 2 mL of the stable consortium and incubated at 37 °C and 125 rpm for 192 h. Samples were taken at regular time intervals (0, 4, 8, 12, 24, 48, 72, 96, 120, 144, 168, 192, 216, 240, and 288 h) to measure the weight of the residual NMP, cell protein content, enzymatic activities, pH (Thermo Orion 0420A1 pH meter), and the concentration of reducing and total sugars in the culture broth. Negative controls consisted of culture media without inoculum. All experiments were conducted in triplicate.

### Evaluation of the degradation rates of NMP

Cultures were harvested by filtration through filter paper (11 µm pore size) under vacuum. Retained solids were dried at 60 °C in a convection oven for 24 h after which the weight of the residual NMP was measured. The NMP degradation (%) was calculated with the following formula:

$$\left[ {\left( {Wt - Wi} \right)/Wt} \right]\, \times \, 100;$$

where *Wt* is total NMP weight before microbial growth; and *Wi* is the residual substrate weight after microbial growth. The degradation rates were calculated dividing the NMP degradation percentage by the time (h after inoculation).

### Determination of the microbial growth

The microbial growth was quantified by measuring the total cellular protein concentration. Culture filtrates (1 mL) were centrifuged at 11,000×*g* and 4 °C for 5 min. The resulting cell pellets were disrupted by incubation with 1 mL of NaOH 500 mM, at 75 °C for 10 min. Cellular lysates were centrifuged at 16,000×*g* and 10 °C for 10 min, and then the supernatant was collected. Cellular lysate protein concentrations were determined using a modified version of the Lowry protein assay (Peterson 1977) using BSA curve (5 to 50 µg) as protein standard.

### Quantification of soluble sugars

During microbial growth, the concentration of reducing sugars in the supernatant was measured using the 3,5-dinitrosalicylic acid (DNS) method (Miller 1959), while the total sugars concentration was determined by the phenol–sulfuric colorimetric assay (DuBois et al. 1956).

### Determination of endo-xylanase and endo-cellulase activities in the culture supernatants

The activities of these hydrolytic enzymes were evaluated in the culture supernatants measuring the amount of reducing sugars released upon substrate hydrolysis by the DNS method. The endo-xylanase activity was assayed at pH 6.5 and 60 °C for 15 min in reactions containing 1.8% (wt/v) xylan from beechwood (Sigma) in phosphate buffer 100 mM. The endo-cellulase activity was measured at pH 4 and 40 °C for 15 min in reactions containing 0.5% (wt/v) carboxymethylcellulose (CMC) (Sigma) in citrate buffer 500 mM. One unit of endo-xylanase or endo-cellulase activity was defined as the amount of enzyme that catalyzed the production of 1 µmol of reducing sugars (xylose or glucose) per minute under the described conditions.

### Compositional analysis of residual NMP after PM-06 growth

Structural carbohydrates and Klason lignin content in the residual NMP were measured by quantitative acid hydrolysis using sulfuric acid (TAPPI T13M method). The monosaccharides obtained from the residual NMP, were quantified by high performance liquid chromatography with refractive index detection (1220 Infinity II LC Systems, Agilent Technologies, US). Separations were carried out over a Phenomenex RPM-Monosaccharide column (00H-0135-K0) at 80 °C, using water as the mobile phase, at 0.6 mL min^−1^ flow rate.

### Scanning electron microscopy (SEM)

The supramolecular morphology of residual NMP particles was determined using a scanning electron microscope (Jeol JSM-6360 LV). For this purpose, samples were coated with gold using a Denton Vacuum Desk II sample metallizer. The images were acquired at 20 kV accelerating voltage.

### Analysis of the bacterial dynamics using denaturing gradient gel electrophoresis (DGGE)

For the analysis of the bacterial dynamics using DGGE, the metagenomic DNA (mgDNA) of the degradation kinetics was extracted. Cultures were filtered through filter paper to eliminate the residual NMP. Cells were recovered after centrifugation of filtrates, at 11,000×*g* for 10 min at room temperature. The cell pellets obtained were washed (twice) with TEN buffer (100 mM Tris-HCl, 50 mM EDTA, 100 mM NaCl, pH 8), to eliminate culture medium residues. The mgDNA was extracted using the methodology reported by Rojas-Herrera et al. (2008). The isolated mgDNA, was utilized as a template to amplify the V3 region of the bacterial 16S rRNA gene through PCR using the previously reported primers GC338f and 518r (Muyzer et al. 1993). Conventional PCR with GoTaq Flexi DNA polymerase (Promega) was performed using 50 ng of mgDNA. PCR conditions included a 94 °C denaturation for 5 min, followed by 40 cycles of 94 °C for 1 min, 60 °C for 1 min, and 72 °C for 1 min. The process concluded with an elongation stage of 72 °C for 10 min. Amplicons were analyzed by DGGE using the DCODE Universal Mutation Detection System (BioRad Laboratories). PCR products were loaded into a 6% polyacrylamide gel with a denaturing gradient urea:formamide of 30 to 70%. Electrophoresis conditions were 70 V for 16 h at 60 °C in 1× tris-acetate-EDTA (TAE) buffer. The gel was stained with SYBR Gold dye (Molecular Probes) and viewed with a Gel Doc XR system photo documentary device (BioRad). Bacterial structure dynamics during degradation was determined using a similarity analysis based on the Jaccard index, followed by unweighted pair-group method analysis (UPGMA) to construct a clustered dendrogram with the BioNumerics software (Applied Maths). Species richness were calculated with the SDR ver. 1.4.2 software (Pisces Conservation LTD) using densitometry data and identified DGGE band counts as input data.

### mgDNA sequencing and data processing

The sequencing of the extracted mgDNA (8 h samples), was carried out at Admera Health Inc. (NJ, USA), using the Illumina NextSeq platform. Output files were processed by Trimmomatic (Bolger et al. 2014) and checked by FastQC suite. Sequencing data, with a Phred quality score ≥ 25, were assembled using the IDBA_UD algorithm (Peng et al. 2012), and aligned with DIAMOND (Buchfink et al. 2014) against the NCBI nr database (e-value threshold of 1e^−5^) for taxonomic classification.

## Results

### Microbial growth, pH and NMP degradation profiles

**Fig. 1:**
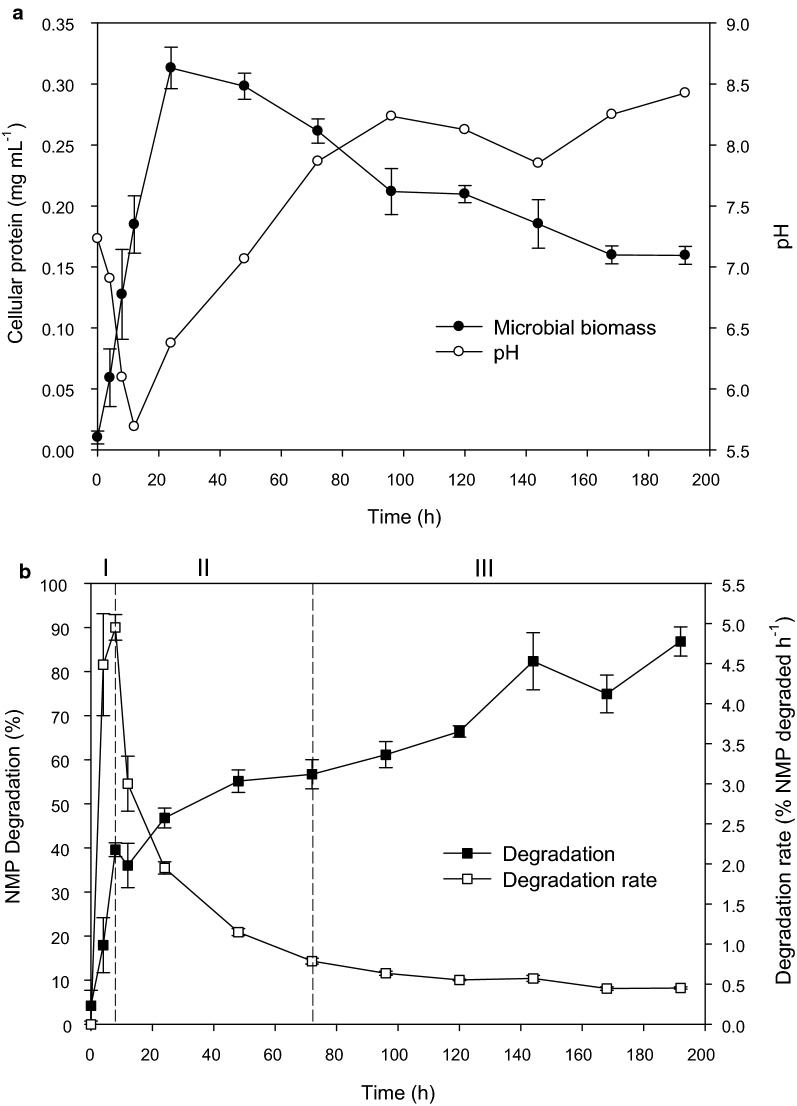
NMP solids consumption, cellular protein concentration and pH profiles. PM-06 growth in terms of cellular protein concentration and pH dynamics (**a**). NMP consumption in terms of the percentage of weight loss and degradation rates (**b**). The NMP degradation stages are indicated with the roman numerals I, II and III on the top of the panel

The consortium PM-06 was obtained after the enrichment and stabilization of the native microbiota present in NMP. The evolution of the microbial growth and pH were analyzed during the degradation of NMP by PM-06 (Fig. 1a). Microbial growth profile showed the absence of a lag phase as consequence of the successive cultivation. Moreover, as result of the microbial metabolism, pH fluctuated along the culture.

The degradation profile of NMP was divided into three stages according to the calculated degradation rates (Fig. 1b). From 0 to 8 h (stage I), the maximum degradation rate occurred (4.95 ± 0.96% of NMP degraded per hour) resulting in around 40% of total consumption. From 8 to 72 h (stage II), the degradation rates decreased and the total percentage of NMP degraded reached 56%. From 72 to 192 h (stage III), the lowest degradation rates were obtained and the maximum percentage of NMP degraded (86.8%) was reached at 192 h. No further changes were observed after this time.

Results indicate the existence of a relationship between the microbial growth, pH and NMP degradation. The stage I is coincident with the early exponential phase of growth and the acidification of the culture medium. During the stage II, the exponential phase ends and the pH started increasing. Finally, in the stage III the protein cell concentration declined and the maximum pH values (close to 8.5) were obtained. It is important to note that culture media contains yeast extract and starch derived from the nixtamalization process; however, results indicate that the consumption of NMP was the main metabolic activity.

### Sugar concentration in the culture supernatant

**Fig. 2:**
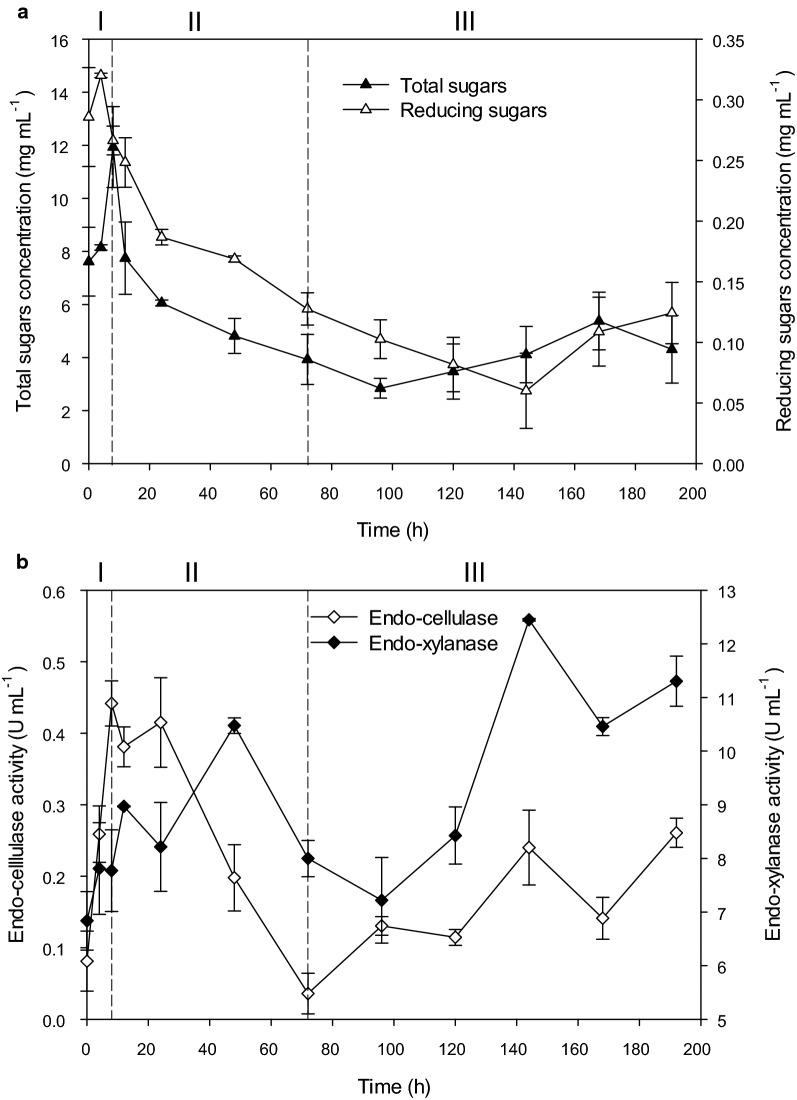
Concentration of soluble sugars and production of enzymatic activities along the degradation of NMP. Total and reducing sugars concentration (**a**). Endo-xylanase and endo-cellulase activities (**b**)

The solubilization of NMP into the medium was determined through the quantification of the total and reducing sugars (Fig. 2a). Total sugars concentrations were one order of magnitude higher than the reducing ones, suggesting the abundance of molecules with high degree of polymerization (DP). During the first 4 h, the concentration of both parameters increased; however, the reducing sugars decreased after this time possibly because include molecules with low DP (mono and oligosaccharides) which can be directly transported into the cytoplasm for further metabolic processing. The stage II was characterized by a decrement in the solubilized sugars, whilst in the stage III a slight increment was observed.

### Production of endo-xylanase and endo-cellulase enzymatic activities

The consortium PM-06 is a prolific endo-xylanase producer, the activities measured were within the interval reported for consortia with high xylanase titles (Zhang et al. 2018), and were one order of magnitude greater than endo-cellulases. Results indicate the presence of differences in the production profile and fluctuations of the activities over time. During the stage I of degradation, both enzymatic activities increased obtaining the maximum value for endo-cellulases at the end of this period (Fig. 2b) The highest endo-xylanase activity was attained in the stage III, at 144 h of growth. Interestingly, a similar behaviour in the production of both activities was observed in the degradation stage III. The fluctuations observed, could be the result of changes in gene expression, proteolysis, and microbial successional events (Bohacz 2018; Jiménez et al. 2018; Li et al. 2017).

### Composition and surface structural analysis of NMP

**Fig. 3:**
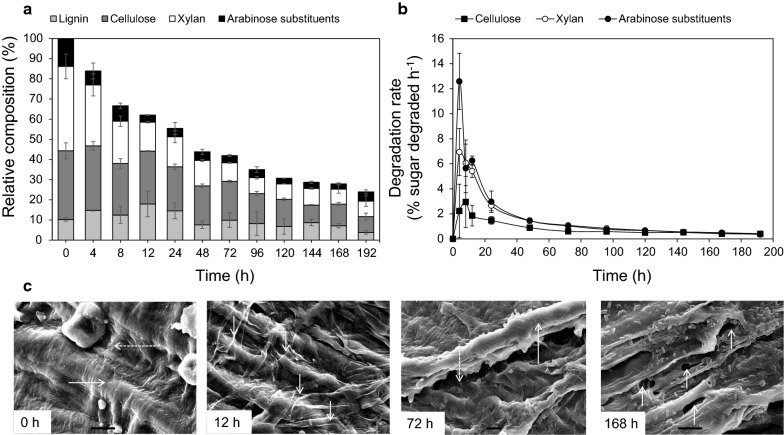
Changes in composition and surface structure of the residual NMP during degradation. Composition of NMP residues during degradation (**a**). Degradation rates of NMP components (**b**). Scanning electron microscopy analysis (**c**). Time 0 h: SEM micrograph of intact NMP (1500×); the continuous horizontal arrow indicate the intact cellulose fiber, while the discontinuous horizontal arrow indicates the intact hemicellulose. 12 h of microbial degradation (1100×): the short down-arrows indicate the hemicellulose layer removal. 72 h of microbial degradation (1000×): the vertical continuous arrows indicate the exposed cellulose fiber while the vertical discontinuous arrows indicate the grooves formed in the internal matrix. 168 h of microbial degradation (1600×): the short up-arrows indicate the pores and cavities formed in the cellulose fibers. Scale bar = 10 µm

Changes in the composition and surface structure of residual NMP were analyzed during the degradation process. The relative composition in terms of cellulose; hemicellulose (xylan and arabinose substituents); and lignin, was determined and presented in Fig. 3a. Results indicate that NMP was sequentially degraded, beginning with the components of the hemicellulosic fraction (Fig. 3a). The highest degradation rates of hemicellulose were observed during stage I; however, at 4 h the arabinose substituents degradation rate was almost twice the xylan rate. The content of arabinose and xylan in NMP decreased around 50% at the end of this lapse of time (Fig. 3b). Cellulose degradation occurred at lower rates, obtaining the highest values also during the stage I (Fig. 3b). The cellulose solubilized in the stage I corresponded to 35% of the total degraded (Fig. 3a). The lignin content along the degradation process showed an erratic behaviour, reaching a degradation around 50% after 48 h of incubation.

Structurally, NMP consists of three zones with a different morphology: zone I is associated with the pedicel; zone II with the embryo; and the zone III covers the endosperm (Gutiérrez-Cortez et al. 2010). This study was focused on the microstructural changes in the surface of the zone III that accounts for the 90% of the intact NMP.

SEM images showed the presence of starch granules, and cellulose and hemicellulose fibers in the surface of NMP prior sterilization (Fig. 3c). The structural characteristics are coincident with the described by Gutiérrez-Cortez et al. (2016), where the integrity of cellulose and hemicellulose structure was maintained after nixtamalization. Sterilization solubilized the starch granules but maintained intact the plant cell wall structure (Additional file 1: Figure S1). After 12 h (stage II), the NMP surface was less structured with the fibers of cellulose more exposed. At 72 h, the plant cell wall structure was loosened with cavities that exposed internal portions of the tissue (Fig. 3c). At the end of the process, the NMP deterioration was deep with fissures, pores and trenches across large areas. The internal tissue was completely exposed allowing the diffusion of enzymes and bacterial cells (Fig. 3c).

### Microbial community dynamics and taxonomy

**Fig. 4:**
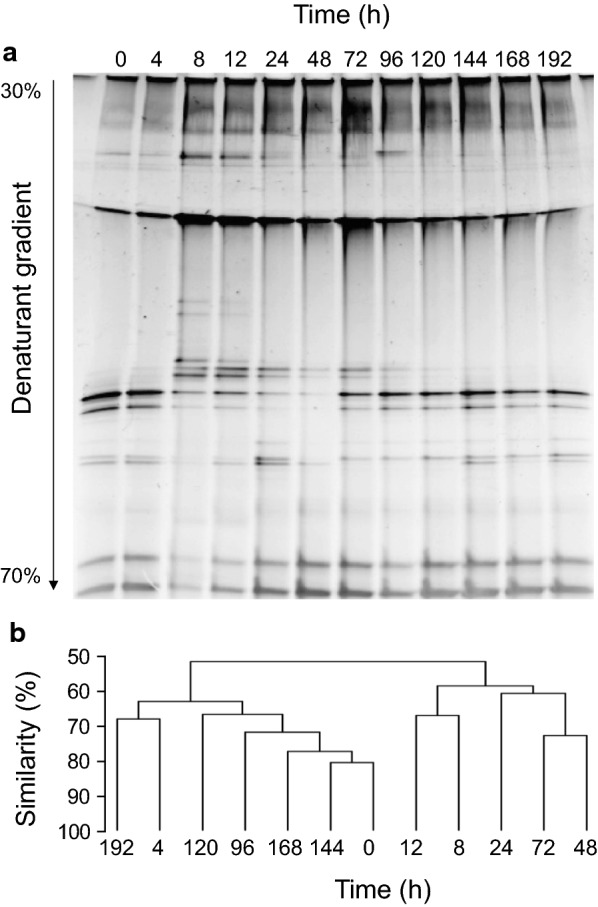
Bacterial population and pH dynamics during NMP degradation. DGGE profile of bacterial 16S rRNA gene amplicons (**a**). UPGMA clustering of DGGE analysis (scale represents similarity from 50 to 100% according to the Jaccard coefficient) (**b**)

The DGGE fingerprints of PM-06 during the NMP degradation, showed variations in the number of operational taxonomic units (OTUs) along the batch of culture (Fig. 4a, Table 1). The number of OTUs (richness) increased during stage I reaching a maximum number at 8 h. In the stage II, the richness started decreasing obtaining the minimum value at 48 h. After this time, the richness became almost constant. The UPGMA dendrogram constructed from DGGE band patterns, showed that samples clustered in two different clades with approximately 50% of similarity (Fig. 4b). Notably, the lanes corresponding to the initial degradation times clustered with the final ones, suggesting a cyclical behaviour.

**Table 1:** Diversity analysis obtained from DGGE pattern related to richness and Shannon index

Sampling times (h)	Richness (n OTUs)	Shannon index (H)
0	11	2.041
4	14	2.31
8	19	2.7
12	15	2.502
24	14	2.405
48	10	1.956
72	13	2.317
96	13	2.23
120	12	2.185
144	13	2.231
168	12	2.168
192	12	2.269

Samples representing the time with the highest degradative activity and OTUs richness (8 h), were analyzed by metagenomics. The assembled metagenome statistics are detailed in Additional file 1: Table S1. A total of 24,879 sequences were taxonomically classified. Taxonomic analysis showed the predominance of the Bacteria domain (99.94%) and within this, the phyla Firmicutes (76.31%) and Actinobacteria (13.66%) were the most abundant. Moreover, 156 bacterial genera were identified (sequence identity ≥ 97%), but only seven presented relative abundances above 1%. The top five species identified in PM-06 were *Aneurinibacillus migulanus* (29%), *Paenibacillus macerans* (26%), *Bacillus coagulans* (15.6%), *Microbacterium* sp. LCT-H2 (11%), and *Bacillus thuringiensis* (4.7%).

## Discussion

Nixtamalization is an ancient procedure created by early Mesoamerican cultures for the transformation of maize into products with high nutritional content and more palatability (Serna-Saldivar 2016). In the global era, nixtamalized products (tortillas, chips, etc.) became popular around the world and have been even frequently included in the diet of astronauts (Serna-Saldivar 2016). Predictions indicate a substantial growth of the nixtamalization industries in the coming years (Future Market Insights 2018). NMP is an abundant residue from the nixtamalization industry, and an important source of fermentable sugars. However, few efforts have been made to harness NMP for the production of commodity chemicals. Arabinoxylooligosaccharides with potential prebiotic and antioxidant activities, as well as gums, have been obtained from the hemicellulosic fraction of NMP, using chemical and physicochemical procedures (Carvajal-Millan et al. 2007; Rostro-Alanís et al. 2014). Attempts to use biological methods are scarce, mainly because usually involve pretreatments followed by the application of enzyme preparations (Rostro-Alanís et al. 2014). The employment of a single biological strategy to overcome the recalcitrance of different lignocellulose matrices is not always efficient because the organization and composition of the main biopolymers (cellulose, hemicellulose and lignin), depend on the species, tissue, and maturity of the plant cell wall (Ciolacu 2018). Moreover, the characteristics of industrial residues depend on the process conditions. In nature, microbial communities accomplish the degradation of a variety of lignocellulosic tissues using degradation strategies that result from the metabolic capabilities of the microbial members, the establishment of ecological interactions, and the structure and composition of the substrate (Cortes-Tolalpa et al. 2016; Carlos et al. 2018). Therefore, understanding the mechanisms used by microbial communities for the degradation of lignocellulose, is crucial for the optimization of processes and the design and production of tailored commercial enzymatic preparations.

NMP degradation, apparently corresponds to an endogenous heterotrophic succession where the abundance of the PM-06 members fluctuates as result of changes in substrate structure, availability of easy metabolizable substrates, microbial interactions, physicochemical conditions of the media, and metabolic versatility of key microorganisms (Fierer et al. 2010; Jiménez et al. 2017). The degradation was sequential with fluctuations in the pH of the media along the process. Stage I was characterized by a fast decrement of NMP solids and pH. The presence of high degradative activities in early stages of culture has been related with the exponential phase, where microorganisms grow rapidly, achieving a fast degradation of lignocellulose (Liang et al. 2017; Zhang et al. 2018). Moreover, literature reports high expression levels of lignocellulolytic enzymes during the first hours of growth (Alessi et al. 2018; Jiménez et al. 2018). Fluctuations in the pH of media along the degradation process is frequently reported in lignocellulose-degrading microbial consortia (Alessi et al. 2018; Hui et al. 2013; Liang et al. 2017; Wongwilaiwalin et al. 2010; Zhang et al. 2018). The acidification has been identified as a consequence of fermentative processes in which microorganisms produce organic acids. Thus, during the exponential phase the PM-06 members acquire energy mainly through the fermentation of sugars obtained from NMP degradation. In this stage, only 35% of cellulose was degraded, a fraction which probably corresponds to the amorphous structural regions. The main sugars released from NMP were arabinose and xylose; however, in the first 4 h of culture, arabinose substituents elimination rate was higher than xylan degradation. In NMP, the xylan backbone of hemicellulose is highly substituted with arabinose and other molecules (Rostro-Alanís et al. 2014) that block the action of endo-xylanases (Agger et al. 2010; Correia et al. 2011). The early enzymatic elimination of arabinose units is a synergistic strategy to facilitate the action of endo-xylanases, promoting the xylan hydrolysis (Agger et al. 2010; Correia et al. 2011; Biely et al. 2016). During the degradation of NMP solids, high DP soluble sugars are released into the medium. These sugars are further saccharified for the production of molecules easily metabolized by microorganisms.

In stage I, the endo-cellulases peaked and 70% of the maximum endo-xylanase activity was present. It is well known the existence of a synergistic relationship between cellulases and xylanases (Hu et al. 2011; Kim et al. 2014; Malgas et al. 2017), that could be favoured by the pH conditions. Although xylanases from *Bacillus* and *Paenibacillus* have demonstrated to be active on a broad pH range (5 to 12) (Mongkorntanyatip et al. 2017; Kurrataa’Yun and Meryandini 2015), the optimal pH interval for the majority of the bacterial endoglucanases is from 5 to 7 (Orencio-Trejo et al. 2016; Sadhu and Maiti 2013). Under these physicochemical conditions, the removal of xylan by endo-xylanases, would allow the access of endo-cellulases to degrade cellulose microfibrils. On the other hand, the degradation of cellulose would allow the access of xylanases to deeper structures. In addition to endo-cellulases and endo-xylanases, other enzymes not measured in this work, like lytic polysaccharide monooxygenase (LPMO), and ligninases, could also enhance the lignocellulose degradation (Bissaro et al. 2018; Hu et al. 2018). The synergistic relationship between enzymes maximize the degradation of cellulose even with low endo-cellulase activity titles. SEM micrographs evidenced the exposition of cellulose fibres after the elimination of hemicellulose from the surface of the residue and the formation of cavities, in further times, that provide access not only to enzymes but also to microorganisms. The degradation process is focused in the elimination of the diffusion barriers for the permeation of water and enzymes.

The degradation of NMP in stage I was faster than the reported for other consortia using different residues (8 h compared to 3 to 6 days), likely as consequence of the NMP structure (Zhang et al. 2018; Liang et al. 2017). Nixtamalization originates changes in the external layers of pericarp such as the loss of cuticle, and formation of pores (Gutierrez et al. 2007; Gutiérrez-Cortez et al. 2010, 2016) that may increase the substrate availability and accessibility of water and enzymes, promoting a fast degradation.

NMP components were consumed at lower rates in stages II and III. Interestingly, in these periods of time the pH of the media was alkalinized. Alkalinization has been related to different metabolic events like organic acids consumption, and the secretion and accumulation of aminoacid catabolic products, between others (Pometto and Crawford 1986; Alessi et al. 2018). In PM-06 cultures, the pH of the medium increased as soon as the concentration of soluble sugars decreased. Some bacteria, like the lactic acid bacteria, response to starvation conditions through the alkalinization of the cytosolic and extracellular pH (Papadimitriou et al. 2016). Alkaline conditions increase the solubility of lignin and cellulose (Pometto and Crawford 1986; Evstigneev 2011), favouring the enzymatic attack. Moreover, alkalinization promoted modifications in the PM-06 bacterial succession inducing probably the growth of microorganism able to produce alkaliphilic enzymes or to degrade aromatic compounds like those present in lignin (Alessi et al. 2018; Carlos et al. 2018).

There is an apparent relationship between the diversity and the capacity of a consortium to degrade lignocellulose (Evans et al. 2017; Jiménez et al. 2017). Therefore, consortia are usually obtained from the enrichment of extremely diverse communities like the present in compost or soil. In contrast, PM-06 microbiome is the product of two selection processes, the first was nixtamalization and the second was the enrichment of the NMP microbiome to obtain the stabilized consortium. However, in this case an efficient lignocellulose-degrader consortium was obtained from of a selected source of microorganisms. As result of the selection conditions, the composition of PM-06 widely differs from the selected native microbiota present in unprocessed maize pericarp natural fermentations, where yeasts and bacteria from the Lactobacillales order were abundant (Decimo et al. 2017). The effect of diversity on the effectiveness of degradation was observed during the process. At 8 h, the maximum values of degradation and microbial diversity and richness were obtained. The increment in diversity provided different and redundant metabolic abilities that in combination produced an efficient process.

Microorganisms present in PM-06 are part of the Bacteria domain and members of the Bacillales and Actinomycetales orders. The most abundant genera were *Bacillus*, *Aneurinibacillus*, *Paenibacillus*, and *Microbacterium*. Several species of *Bacillus* and *Paenibacillus* are recognized as great plant biomass degraders (Ghio et al. 2018; Orencio-Trejo et al. 2016). These microorganisms synthesize lignocellulolytic enzymes active under different pH and temperature conditions (Gao et al. 2018; Gastelum-Arellanez et al. 2014; Zhang et al. 2017). The genus *Aneurinibacillus* is part of the ruminal microbiota able to synthesize enzymes that work on lignocellulose (Asem et al. 2017). In contrast, *Microbacterium* species contain few genes encoding for lignocellulolytic enzymes; however, possess a great metabolic potential to degrade arabinoxylans (Yeager et al. 2017). Bacillales and Actinomycetales are frequently present in plant biomass-degrading consortia and different studies suggest a synergistic relationship between them (Azizi-Shotorkhoft et al. 2016; Puentes-Téllez and Salles 2018; Ventorino et al. 2015). In particular, the genus *Microbacterium* together with Bacillales such as *Paenibacillus* or *Bacillus* have demonstrated to be the key bacteria of synthetic lignocellulose-degrading minimal consortia (Jiménez et al. 2018; Azizi et al. 2018). Metagenomic data would allow to predict the roles of microbial species in degradation; however, the ecological relationships define the specific participation of each member. Thus, a metatranscriptomic and secretomic analysis are necessary to unveil the enzyme producers along degradation.

The microbial population of PM-06 presented a cyclical behaviour with the existence of a minimum set of bacterial species possibly conformed by the *Aneurinibacillus*, *Bacillus*, *Paenibacillus*, and *Microbacterium* genera, whose presence and ecological relationships would be important to preserve the consortium viability and degradation capacity (De Lima Brossi et al. 2016; Lee et al. 2013; Puentes-Téllez and Salles 2018).

Consortium PM-06 is an efficient NMP degrader and a prolific endo-xylanase producer. The mechanism utilized for NMP degradation was sequential where the substrate structure, microbial growth, pH, synergistic relation between enzymes, ecological relationships, and microbial dynamics were intimate related. All the tools generated by the consortium worked coordinated to increase the substrate availability through the solubilization of components and elimination of structural diffusion barriers. More studies are necessary to analyze the whole set of enzymes produced, the effect of pH on the enzyme activity and stability, gene regulation strategies, and changes in NMP permeation along time, to obtain specific information of the mechanism. This is the first report about the degradation of NMP using a microbial consortium.

## Additional file

**Additional file 1: Table S1.** Metrics of assembled metagenome. **Figure S1.** SEM Micrograph of nixtamalized maize pericarp surface morphology after sterilization by autoclaving at 121 °C for 15 min.

## Data Availability

The metagenome analyzed in this article is available in NCBI portal with the Bio Project ID PRJNA522744 (Biosample accession: SAMN10953775).

## Abbreviations

CMC: carboxymethylcellulose
DGGE: denaturant gradient gel electrophoresis
DNS: 3,5-dinitrosalicylic acid
DP: degree of polymerization
NMP: nixtamalized maize pericarp
SEM: scanning electron microscopy
OTUs: operative taxonomic units
