# Supplementary material for: Degradation profile of nixtamalized maize pericarp by the action of the microbial consortium PM-06
Source: AMB Express. 2019 Jun 13;9:85. doi: 10.1186/s13568-019-0812-7 (PMC6565776; doi:10.1186/s13568-019-0812-7)
Supplement: Supplementary file 1 — Additional file 1: Table S1. Metrics of assembled metagenome. Figure S1. SEM Micrograph of nixtamalized maize pericarp surface morphology after sterilization by autoclaving at 121 °C for 15 min. [file 13568_2019_812_MOESM1_ESM.docx]

**AMB Express**

Additional file 1

**Degradation profile of nixtamalized maize pericarp by the action of the microbial consortium PM-06**

José Germán Serrano-Gamboa ^1^, Rafael Antonio Rojas-Herrera ^1^, Araceli González-Burgos ^2^, Jorge Luis Folch-Mallol ^2^, Diego Javier Jiménez ^3^ and Mónica Noel Sánchez-González ^1^*

^1^ Facultad de Ingeniería Química, Universidad Autónoma de Yucatán, Mérida, México.

^2^ Centro de Investigación en Biotecnología, Universidad Autónoma del Estado de Morelos, Cuernavaca, México.

^3^ Microbiomes and Bioenergy Research Group, Department of Biological Sciences, Universidad de los Andes, Bogotá, Colombia.

*Correspondence

Name: Mónica Noel Sánchez-González

Email: [monica.sanchez@correo.uady.mx](mailto:monica.sanchez@correo.uady.mx)

**Table S1.** Metrics of assembled metagenome.

| Bases sequenced | 25958302 |
| --- | --- |
| Contigs | 592 |
| Smallest | 1006 |
| Largest | 570693 |
| Average length | 43848.5 |
| Taxonomic annotated sequences | 24879 |





**Figure S1.** SEM Micrograph of nixtamalized maize pericarp surface morphology after sterilization by autoclaving at 121 °C for 15 min.
